# Exploring the effect of teacher autonomy support on Chinese EFL undergraduates’ academic English speaking performance through the mediation of basic psychological needs and classroom engagement

**DOI:** 10.3389/fpsyg.2024.1323713

**Published:** 2024-02-20

**Authors:** Yanning Wang, Weihua Luo, Xian Liao, Pengfei Zhao

**Affiliations:** ^1^School of Foreign Languages, Dalian Maritime University, Dalian, China; ^2^Department of Chinese Language Studies, The Education University of Hong Kong, Tai Po, Hong Kong SAR, China

**Keywords:** teacher autonomy support, basic psychological needs, classroom engagement, academic English speaking, Chinese EFL undergraduates

## Abstract

**Introduction:**

The capacity to speak English for academic purposes is a pivotal facet of language education and assessment. Despite the substantial research approving the significant role of teachers in L2 learning, it remains unclear how exactly teachers’ support for students’ learning autonomy influences EFL learners’ academic English speaking performance.

**Methods:**

To address this primary concern, this study drew ground from the Self-System Model of Motivational Development (SSMMD) and adopted a mixed-method approach to examine teacher autonomy support’s direct and indirect effects on Chinese EFL undergraduates’ academic English speaking performance through the mediation of basic psychological needs and classroom engagement. 247 first-year university students participating in academic English speaking courses were recruited in this study.

**Results and discussion:**

The quantitative results of the questionnaire indicated that students’ perceived teacher support for autonomy directly predicted English speaking performance, and it also indirectly influenced students’ speaking performance via the mediation of classroom engagement and basic psychological needs. Students’ responses in the semi-structured interview further verified the positive effect of teacher autonomy support on academic English speaking development in the classroom. Pedagogical implications were also discussed based on the findings.

## 1 Introduction

In the globalized world where English is a *lingua franca*, English speaking skills are critically important for university students to orally communicate ideas, master disciplinary knowledge, and engage in the academic community during their academic learning in higher education (Peng and Woodrow, 2010; Zhang et al., 2020; Pitura, 2022). It has been acknowledged that the learning context, especially teachers’ support for students’ learning, plays a significant role in students’ acquisition of communicative abilities in the L2 classroom (Zarrinabadi et al., 2022; Solhi, 2023). The primary aim of teacher support is to cultivate students to be autonomous language learners (Borg and Alshumaimeri, 2019). Grounded in self-determination theory (SDT), teacher autonomy support is an important type of teacher support (Lietaert et al., 2015), which facilitates learning by empathizing with learners, acknowledging their emotions, offering necessary information, and minimizing pressures (Zarrinabadi et al., 2022). Theoretically speaking, autonomy-supportive teachers can effectively motivate students by catering to their needs, interests, and preferences, which is essential for fostering engagement (Vansteenkiste et al., 2005). In addition, some researchers claimed that students’ psychological factors and engagement are highly context-dependent (e.g., Hiver et al., 2021). In such a supportive learning environment, students may inherently perceive themselves as a valuable member of a learning community (e.g., L2 classroom) and thus become more intrinsically motivated with autonomous engagement in L2 speaking endeavors such as seeking opportunities to practice their speaking skills or express their opinions confidently (e.g., Alrabai, 2021; Wu and Hung, 2022; Solhi, 2023). Despite the existing insights, few empirical studies have focused on how exactly the teachers’ autonomy support influences L2 speaking performance via students’ basic psychological needs and engagement.

Furthermore, this research issue deserves attention in the Chinese educational context, where English is a core subject in higher education and a medium of instruction in various disciplines (Tong and Shi, 2012). Nevertheless, due to the prevalence of an exam-oriented approach to L2 teaching, students’ oral proficiency in English is still unsatisfactory compared with their written skills (Gu et al., 2022). Moreover, influenced by the traditional Confucian culture which prioritizes golden silence and places high respect for teachers’ authority, Chinese students are usually reluctant to speak out their ideas actively in class (Chen et al., 2022). Nonetheless, when students enter university, they are expected to become self-regulated learners with high autonomy during their academic pursuits, including English speaking skills, which particularly requires students’ active participation and open communication (Zhang et al., 2020). Therefore, conducting research in the context of Chinese higher education provides a unique oriental perspective that contributes to the existing body of literature on L2 speaking.

## 2 Literature review

### 2.1 Theoretical framework

**Figure 1:**
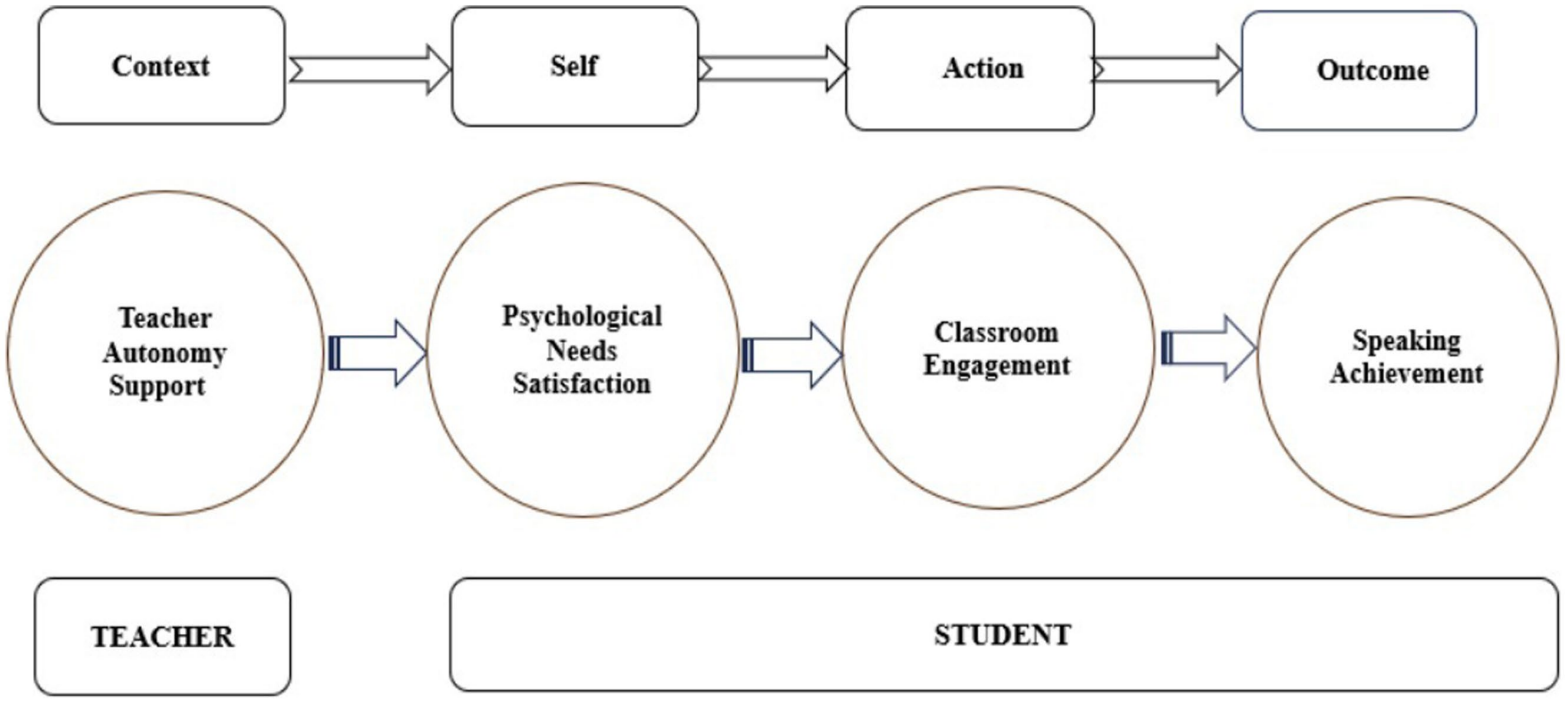
The Adapted Self-System Model of Motivational Development (SSMMD) (Connell and Wellborn, 1991; Skinner et al., 2009).

This study was underpinned by the Self-System Model of Motivational Development (SSMMD), which originated from SDT, which postulated that students’ self-system is related to the social context, engagement, and various outcomes in the general educational context (Connell and Wellborn, 1991). SSMMD involves four influential components: context, self, action, and outcome. The context factor pertained to the social environment of learners, especially teacher support to students’ learning. The self factor encompasses learners’ beliefs, values, attitudes, and perceptions of basic psychological needs. The engagement factor primarily focused on conceptualized engagement under the classroom context with foci on students’ cognitive, behavioral, agentic, and emotional involvement. The above three important interrelated factors may affect students’ learning outcomes. As is found by Reeve (2013), teaching context could predict self-processes (i.e., need satisfaction), which in turn predicts engagement, ultimately exerting influence on academic achievement. In L2 research, abundant evidence also indicated that teachers provide more effective support to students to satisfy their learning needs (e.g., autonomy, competence, and relatedness), and students are more likely to engage themselves in their language learning activities actively, thus contributing to higher L2 achievement (Oga-Baldwin et al., 2017; Dincer et al., 2019; Noels et al., 2019; Karbakhsh and Ahmadi Safa, 2020; Hiver et al., 2021). Based on these articulations, we proposed that in the L2 English speaking classroom, students who positively perceived the learning support from their teachers may feel more satisfied psychologically, in turn, engaged more in the speaking classroom, and finally achieve higher L2 speaking proficiency (see Figure 1).

### 2.2 Basic psychological needs in language learning

According to SDT, basic psychological needs (BPN) include autonomy, competence, and relatedness as the fundamental components for individuals’ integration, growth, and healthy development (Ryan and Deci, 2017). Autonomy pertains to learners’ ability to organize and regulate their behavior independently while making continuing efforts to accomplish learning objectives (Ryan and Deci, 2000). Competence signifies a condition in which the learner perceives effectiveness, skill, and the ability to carry out designated tasks proficiently. To foster a sense of competence, it is essential to establish a clear framework and learning objectives right from the outset of their language learning endeavors (Ryan and Deci, 2000). Finally, relatedness refers to connectedness with other people and developing a sense of warmth with others. It has been suggested that the psychological need for relatedness is essential for the internalization of learning processes as well as for taking ownership of learning and engagement (Ryan et al., 1995).

The importance of basic psychological needs in academic learning has been supported in previous literature (e.g., Joe et al., 2017; Zhen et al., 2017; Karbakhsh and Ahmadi Safa, 2020; Alamer, 2022). For instance, Zhen et al. (2017) found that Chinese middle school students’ basic psychological needs (e.g., relatedness) significantly predicted their engagement in academic learning. The association between basic psychological needs and engagement was also discussed in L2 literature. For example, Karbakhsh and Ahmadi Safa (2020) examine the relationship between several key individual difference factors of L2 achievement among Iranian EFL learners (e.g., basic psychological needs, self-efficacy, goal orientation, language learning strategies, etc.). They concluded that students’ basic psychological needs did not directly predict final L2 achievement but could indirectly influence achievement through their motivational involvement (i.e., goal orientations). Similarly, Joe et al. (2017) investigated the critical individual and situational factors (i.e., basic psychological needs and class climate) behind Korean secondary school students’ L2 performance. They found that students’ basic psychological needs not only held a significant directive prediction on the intended engagement in communication (e.g., willingness to communicate) but also predicted the L2 achievement via the mediation of willingness to communicate. Based on previous evidence, we can assume that students’ internal psychological needs may indirectly influence L2 learning outcomes through other psychological factors, such as motivation and emotion, which largely overlap students’ cognitive and emotional engagement (Alamer and Lee, 2021; Alamer, 2022).

However, Alamer (2022) examined 215 Saudi EFL learners’ basic psychological needs in online language learning during the COVID-19 pandemic and indicated that students’ basic psychological needs did not predict their willingness to continue engaging in English learning. Extant findings on the linkage between basic psychological needs and engagement appear mixed; thus, we need more empirical evidence to clarify the relationship in the EFL-speaking context. It is also critically important to specify whether EFL students’ basic psychological needs in speaking learning influence their English speaking performance through learning engagement.

### 2.3 Classroom engagement in language learning

Educators increasingly acknowledge the importance of engagement in language learning (Mercer and Dörnyei, 2020; Zhao et al., 2023; Zhu et al., 2023). There has been a consensus that engagement is a multidimensional construct, including cognitive, behavioral, and emotional engagement (Fredricks et al., 2004; Reeve, 2012; van Uden et al., 2014). As some researchers critiqued (Reeve and Tseng, 2011), the existing three-dimensional engagement model fails to consider learners’ active and constructive contributions to their learning activities, termed agentic engagement. It has been considered another feasible component in the previous framework (e.g., Eccles, 2016). This study attempted to follow the more comprehensive engagement model to examine its role in academic English speaking learning. In language learning, engagement is the most proximal predictor of students’ learning outcomes (e.g., Noels et al., 2019; Zhang et al., 2020; Hiver et al., 2021). For instance, Dincer et al. (2019) found that EFL students’ emotional engagement positively predicted their language achievement. Likewise, Zhang et al. (2020) examined the influential contributors to Chinese undergraduates’ EFL listening and speaking performance. They discovered the significant prediction of classroom engagement on students’ final English listening and speaking grades. In addition to functioning as a predictor of learning outcomes, engagement also acted as a mediator between motivational variables and performance (Skinner et al., 2008; Zhao et al., 2023).

As Skinner et al. (2008) postulated in their model, students who perceived teachers’ support positively were more likely to feel satisfied psychologically and then engage more in their learning, ultimately achieving higher attainment of learning. Previous studies on EFL learning also support these assumptions (e.g., Dincer et al., 2019; Lee et al., 2022). Dincer et al. (2019) showed that academic English learners who received positive teacher autonomy support were more likely to demonstrate a higher sense of satisfaction and active involvement in the language classroom, thereby increasing the likelihood of obtaining higher grades. Based on a large-scale investigation of English speaking learning among Chinese university students, Peng and Woodrow (2010) established that teacher support indirectly predicted students’ engagement in speaking tasks by mediating self-determined perception of their competence (one of the basic psychological needs). Lee et al. (2022) also suggested that students with greater autonomy in choosing their favorite speaking tasks would experience greater psychological satisfaction and thus improve their learning engagement.

### 2.4 Teacher autonomy support in language learning

Teacher autonomy support refers to the instructional strategies employed to create a classroom setting and teacher-student relationship that foster students’ need for autonomy (Reeve, 2016). Providing students with autonomy offers them more flexibility in choosing their learning objectives, potentially leading to heightened cognitive engagement (Lee et al., 2022). For instance, Vansteenkiste et al. (2005) compared 140 first-year undergraduates who received autonomy support from their teachers with the control group and revealed that the former obtained increased need satisfaction, greater autonomous motivation, enhanced classroom engagement, and higher academic achievement.

Teacher autonomy support is also essential for successful language learning (Deci et al., 1994; McEown et al., 2014; Lietaert et al., 2015; Mackey and Gass, 2015; Wang et al., 2017). For instance, McEown et al. (2014) discovered that 128 Canadian undergraduates experienced higher levels of performance and engagement in L2 classrooms when they received sufficient teacher support compared to those who perceived less support from teachers. Likewise, Lietaert et al. (2015) revealed that teachers’ autonomy support was a proactive factor for secondary male students’ engagement in the Dutch language classroom. In addition to engagement, Dincer et al. (2019) investigated the associations between teacher autonomy support, basic psychological needs, classroom engagement, and language proficiency among 412 Turkish EFL university students. They suggested that teacher autonomy support significantly predicted students’ basic psychological needs (including autonomy, relatedness, and competence) and their attendance in L2 class rather than general L2 performance. More recently, a study by Zarrinabadi et al. (2022), who examined 392 Iranian undergraduates’ oral language learning, showed that students’ perceived teacher autonomy support positively predicted their perceived L2 English communicative competence and willingness to communicate. In the existing literature, however, the role of teacher autonomy support in the process of academic English speaking acquisition among EFL learners remains an unexplored area.

**Figure 2:**
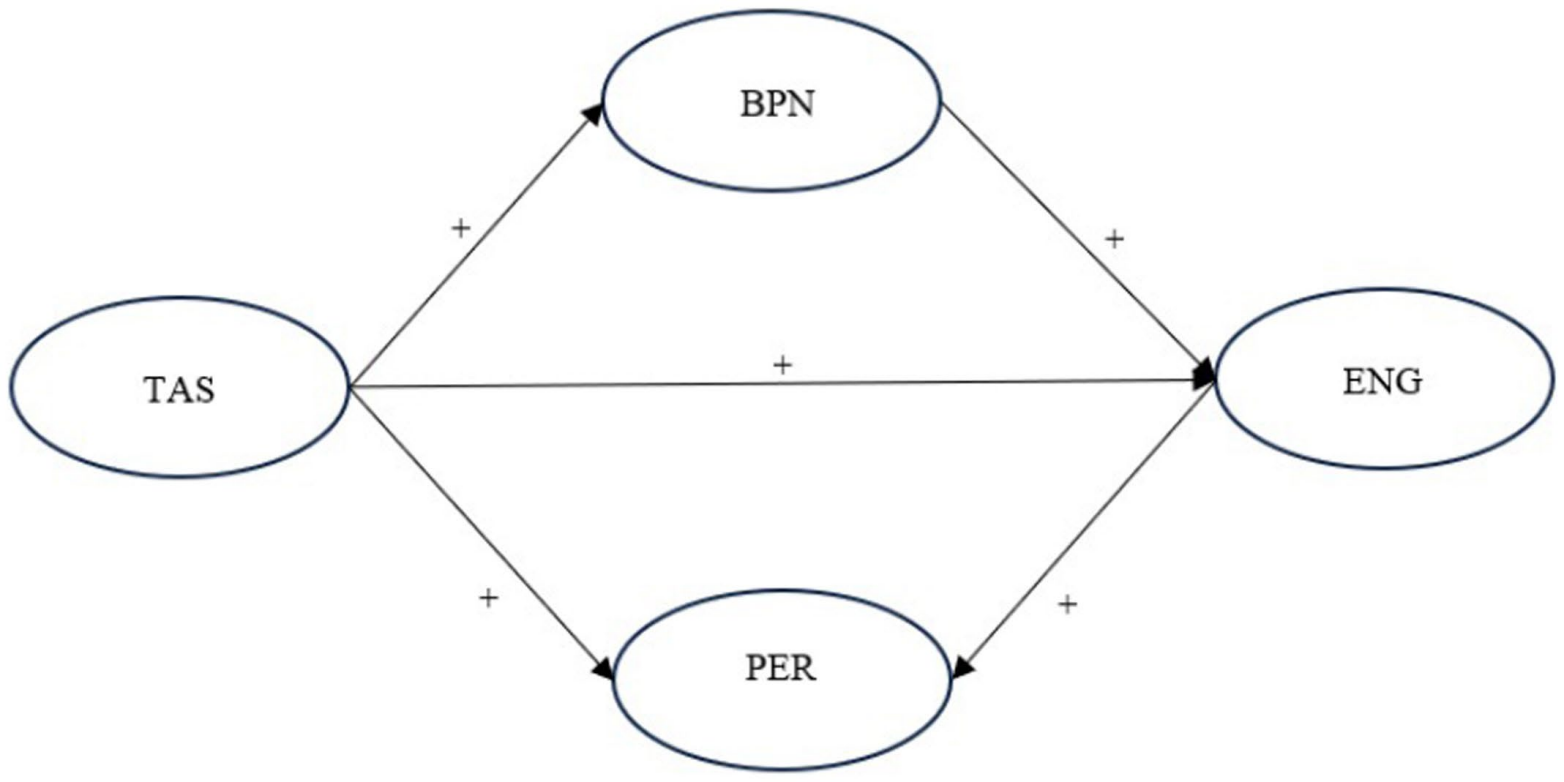
The hypothesized model. TAS refers to teacher autonomy support; BPN refers to basic psychological needs; ENG refers to classroom engagement; PER refers to academic speaking performance.

Based on the theoretical knowledge and empirical evidence, we hypothesize that teacher autonomy support can predict EFL students’ English speaking performance through a serial mediation of basic psychological needs and classroom engagement. Thus, we can see how teacher support influences EFL learners’ speaking development (see Figure 2). However, relevant studies are still very limited to our best knowledge; it is therefore essentially necessary to conduct this research.

Correspondingly, two research questions are proposed in this research.

1. What is the direct effect of teacher autonomy support on basic psychological needs, classroom engagement, and academic English speaking performance?
2. What are the indirect effects of teacher autonomy support on academic English speaking performance via basic psychological needs and classroom engagement?

## 3 Methods

A mixed-method explanatory sequential design was adopted to comprehensively understand teacher autonomy support’s direct and indirect effects on students’ English speaking performance. According to Creswell and Plano Clark (2011), the purpose of the quantitative phase in this research was to generally depict the relationships among teacher autonomy support, basic psychological needs, classroom engagement, and academic English speaking performance, while the analysis of interview responses aimed to obtain an in-depth understanding of how exactly subcomponents of teacher autonomy support impact L2 speaking through the three dimensions of basic psychological needs and classroom engagement.

### 3.1 Participants

247 students voluntarily participated in this study, including 185 males and 62 females (*M* = 18.62, *SD* = 1.17). They were from a state-owned university in Northeastern China. To enhance the internationalization of higher education, the university delivered academic English speaking courses to first-year university students majoring in engineering. Within this institute, characteristic of Sino-US joint cultivation, first-year students were required to complete two semesters of academic English speaking training in preparation for English Medium Instruction (EMI) of academic study in the second academic year. Each semester, students had 12 academic speaking classes (90 min per class) where they learned academic speaking skills to qualify for the benchmark score (6.5) of IELTS. In the second term, students received specific instructions on Part 3 of the IELTS speaking subtest before participating in the present study. There are five academic English teachers with 29 to 32 students in each class. We conducted data collection in the second semester.

### 3.2 Instruments

This study employed four instruments: (a) a questionnaire, (b) an academic English speaking task, (c) a scoring rubric for English speaking performance, and (d) an interview.

#### 3.2.1 Questionnaire

The questionnaire consisted of two sections. The first section collected participants’ background information, including gender, age, and college entrance examination scores in English; the second section measured students’ perceived teacher autonomy support, basic psychological needs, and classroom engagement, respectively.

To be specific, the 20-item teacher autonomy support scale was adapted from Awang-Hashim et al. (2017), including four sub-scales: responsibility (3 items, *α* = 0.84), friendliness (7 items, *α* = 0.88), respect (5 items, *α* = 0.84), and confidence (5 items, *α* = 0.87). Example statements included: “*Teacher gives extra attention to weak students*.” A 5-point Likert scale was used, ranging from 1 (strongly disagree) to 5 (strongly agree). The construct validity of this measure was also approved through CFA [*χ*^2^/*df* = 1.89, *p* = 0.00, CFI = 0.96, TLI = 0.95, RMSEA = 0.06].

The 12-item basic psychological needs of the second language scale were adapted from Alamer’s (2022) study, including three components: autonomy (4 items, *α* = 0.77), competence (4 items, α = 0.87), and relatedness (4 items, *α* = 0.89), for example, “*I am able to freely decide my own pace of academic English speaking learning.*” A 7-point Likert-type scale was adopted, ranging from 1 (strongly disagree) to 7 (strongly agree). The construct validity of this measure was also approved through CFA [*χ*^2^/*df* = 2.50, *p* = 0.00, CFI = 0.95, TLI = 0.93, RMSEA = 0.078].

The 22-item classroom engagement scale was adapted from Skinner et al.’s (2009) and Reeve and Tseng (2011) studies. This scale included four components: behavioral (5 items, *α* = 0.88), cognitive (8 items, *α* = 0.90), emotional (4 items, *α* = 0.87), and agentic engagement (5 items, *α* = 0.87). A 7-point Likert-type scale was used, ranging from 1 (strongly disagree) to 7 (strongly agree). The construct validity of this measure was also approved through CFA [*χ*^2^/*df* = 2.40, *p* = 0.00, CFI = 0.92, TLI = 0.90, RMSEA = 0.076]. As the 22 original questionnaire items pertained to English learning in broad terms, to suit the current research context, we made a few modifications by clarifying the context of “academic English speaking”, for example, “*I work hard when we start something new in academic English speaking class*.”

#### 3.2.2 Academic English speaking task

The assessment questions for this task were adapted from Part Three of the International English Language Testing System’s (IELTS) speaking sub-tests for academic purposes (IELTS, 2017). These question items were selected from authentic IELTS tests following discussions with and approval from five front-line teachers who have taught IELTS for more than five years. The primary reason for choosing Part Three was that it provided examiners with a better indication of actual academic speaking performance and score (Quaid, 2018). Example questions include: “*What challenges do teenagers face in today’s society?*,” “*How important is it for teenagers to have a strong sense of identity?*” and “*What advice would you give to teenagers on making the most of their teenage years?*.” Students were required to express and justify their opinions, analyze, discuss, and speculate on the topic in depth. Each participant took around 4 min to complete the speaking task.

#### 3.2.3 Scoring rubric of academic English speaking performance

This study adopted the well-established scoring rubric for the IELTS speaking test, involving fluency and coherence, lexical resource, grammatical range and accuracy, and pronunciation (IELTS, 2017). Each aspect comprised ten levels (0–9), with one point awarded per level. Students’ final scores were the average of the scores of four aspects. For instance, if a student scores 7 in fluency and coherence, 8 in pronunciation, 7 in lexical resource, and 6 in grammatical range, the total score would be calculated as (7 + 8 + 7 + 6) / 4 = 7.

#### 3.2.4 Semi-structured interviews

To further explore the associations among these target variables, the semi-structured interview was adopted. Before the semi-structured interviews, we conducted an one-hour pilot interview with two students who were randomly selected from the participants. This step was essential to ensure the appropriateness of the interview questions, informed by the students’ feedback. The finalized interview outline comprised 16 questions, meticulously aligned with the study’s research questions. These were aimed at delving deeper into the relationships between students’ different perceptions of teacher autonomy support, the various dimensions of basic psychological needs, classroom engagement, and academic English speaking performance, as well as gathering their ideas for the design of supportive speaking courses. Example questions included: “*Do you feel psychologically satisfied regarding autonomy, relatedness, and competence during classroom activities*? *What role does your speaking teacher play in this process?*”

## 4 Procedure

### 4.1 Data collection

Initially, participants performed the speaking test individually in the classroom. To simulate an authentic assessment environment, students were seated and awaited the examiners’ questions. The questions were displayed on a monitor, and participants were asked to articulate their thoughts on the given topics. Following a 10-min break after the tests, students completed the questionnaire using *Wenjuanxing*,^1^ a popular online questionnaire platform in China. They were encouraged to request clarifications from teachers if they encountered any confusion about the questionnaire items.

Within one week, the interviews were conducted. Participants were categorized into two groups (high-TAS and low-TAS) based on their levels of perceived teacher autonomy support, using a two-step cluster analysis. Twelve students, six from each group, were randomly selected to participate in the interviews, which they did voluntarily. The semi-structured interviews were conducted over two consecutive days. The interviews were conducted in an online meeting environment by using Tencent Meeting, a versatile and user-friendly video conferencing application in China. The first author, as the interviewer, conducted the interviews, during which students’ native language (Chinese) was employed for easy communication. At the beginning of each interview, the interviewer introduced herself and outlined the research objectives. This was followed by warm-up questions to acclimatize students to the interview setting. Subsequently, the interviewer posed queries according to the question guide and students’ questionnaire responses. Participants were encouraged to freely express their perceptions of the teachers, the speaking courses, and their engagement in English speaking. Each interview lasted approximately 45 min. All interviews were recorded for subsequent qualitative analysis.

Finally, the scoring of students’ speaking test performances was undertaken by two IELTS trainers, who also served as course lecturers. Both trainers possess over five years of experience in teaching English speaking. Before the scoring process, they were required to acquaint themselves with the marking scheme thoroughly. A preliminary trial scoring session was then conducted on 30 randomly selected audio recordings to address any discrepancies. This involved discussions among raters and researchers until a consensus was reached. Following this, the formal scoring phase commenced, during which the two raters independently evaluated the remaining recordings. The inter-rater reliability was confirmed to be acceptable (*α* = 0.849).

### 4.2 Data analysis

The data collected were analyzed using SPSS 27.0 for statistical computations. Initially, descriptive statistics, including mean, standard deviation, and assessment of normal distribution, were computed to understand the sample characteristics. This was followed by a correlational analysis to explore potential relationships between the target variables. Subsequently, Mplus 8.0 was employed for a two-step structural equation modeling (SEM) approach. This aimed to delineate the direct and indirect effects of teacher autonomy support on students’ academic English speaking performance, mediated by basic psychological needs and classroom engagement. In the first step, confirmatory factor analysis (CFA) was used to assess the measurement model. Modification indices (MI) were examined. Any parameter with MI > 4 that could be theoretically justified would be added to the measurement model to improve the model fit. In the second step, SEM was utilized to examine the structural relationships among the variables of interest, provided that the measurement model exhibited an acceptable fit.

Teacher autonomy support (TAS) was treated as the independent variable, while basic psychological needs (BPN), classroom engagement (ENG), and academic English speaking performance (PER) were considered dependent variables. Maximum likelihood method (MLM) estimator was employed to estimate the parameters. Model fit was assessed using several indices: the Chi-square statistic (*χ^2^*), comparative fit index (CFI), Tucker-Lewis Index (TLI), root mean square error of approximation (RMSEA), and standardized root-mean-square residual (SRMR). To indicate a good model fit, CFI and TLI values above 0.90 and RMSEA and SRMR values below 0.08 were considered acceptable (Kline, 2016). However, to account for the hierarchical structure of our data (students within five teachers’ classes), we used the TYPE = COMPLEX command (Cheong et al., 2023). We checked the model’s fit with several indices: chi-square (*χ^2^*), Comparative Fit Index (CFI > 0.95), Root Mean Square Error of Approximation (RMSEA <0.06), and Standardized Root Mean Square Residual (SRMR <0.05) (Kline, 2016). The indirect effects were evaluated using a 95% bias-corrected bootstrap confidence interval (BCCI) with 2,000 bootstrap samples. Indirect effects were deemed significant if the 95% BCCI was omitted.

The authors were responsible for analyzing students’ interview responses by using Nvivo 11. Initially, the data was subjected to meticulous line-by-line coding, which was then followed by a thorough re-examination to discern the interconnectedness of emerging themes (Creswell, 2014). Our principal aim was to identify specific keywords and phrases that directly correlated with various sub-dimensions of the pertinent constructs (Yao et al., 2023). For instance, expressions such as “*more time devoted to practicing pronunciation*” and “*active participation in group tasks*” were categorized under the sub-dimension of behavioral engagement. Similarly, phrases like “*ability to organize speaking tasks on my own*” and “*regulating my efforts of building up IELTS vocabulary through the learning application*” were classified under autonomy, a sub-dimension of basic psychological needs. Additionally, “*offering personalized feedback and suggestions on assignments*” and “*adequate teacher-student interaction*” were linked to responsibility, a facet of teacher autonomy support. We thus delved into the interplay between these constructs among two student groups. To ensure intercoder reliability, we assigned codes to the entire dataset collectively and repeated the coding process, discussing any discrepancies to reach a consensus (Song and Cho, 2018).

## 5 Results

### 5.1 Descriptive statistics of the variables

Table 1 shows the descriptive statistics and correlations of measured variables. According to the mean value of the TAS (*M* = 3.94, *SD* = 0.66), BPN (*M* = 4.98, *SD* = 0.68), and ENG (*M* = 5.06, *SD* = 0.67), in general, students perceived their academic English speaking teachers as autonomy-supportive and their speaking classroom engaging. They reported moderate satisfaction with their basic psychological needs with their speaking courses. According to the correlational results, the TAS, BPN, ENG, and PER are significantly correlated with each other (*r*_TAS-BPN_ = 0.43, *p* < 0.001; *r*_TAS_-_ENG_ = 0.15, *p* < 0.01; *r*_BPN_-_ENG_ = 0.76, *p* < 0.001; *r*_ENG_-_PER_ = 0.42, *p* < 0.001; *r*_TAS_-_PER_ = 0.21, *p* < 0.01, respectively).

**Table 1:** Descriptive statistics and correlations of measures.

Variables	Mean	SD	Max	Min	Kurtosis	Skewness	1	2	3	4
1.TAS	3.94	0.66	5.0	2.4	−0.979	−0.310	1.00			
2.BPN	4.98	0.68	7.0	3.0	0.205	0.060	0.50^**^	1.00		
3.ENG	5.06	0.67	6.7	3.0	−0.176	0.135	0.38^**^	0.72^**^	1.00	
4. PER	4.93	0.62	6.5	3.0	0.051	0.122	0.39**	0.45^**^	0.51^**^	1.00

*p < 0.05; **p < 0.01.

### 5.2 The relationships between teacher autonomy support, basic psychological needs, classroom engagement, and academic English speaking performance

**Figure 3:**
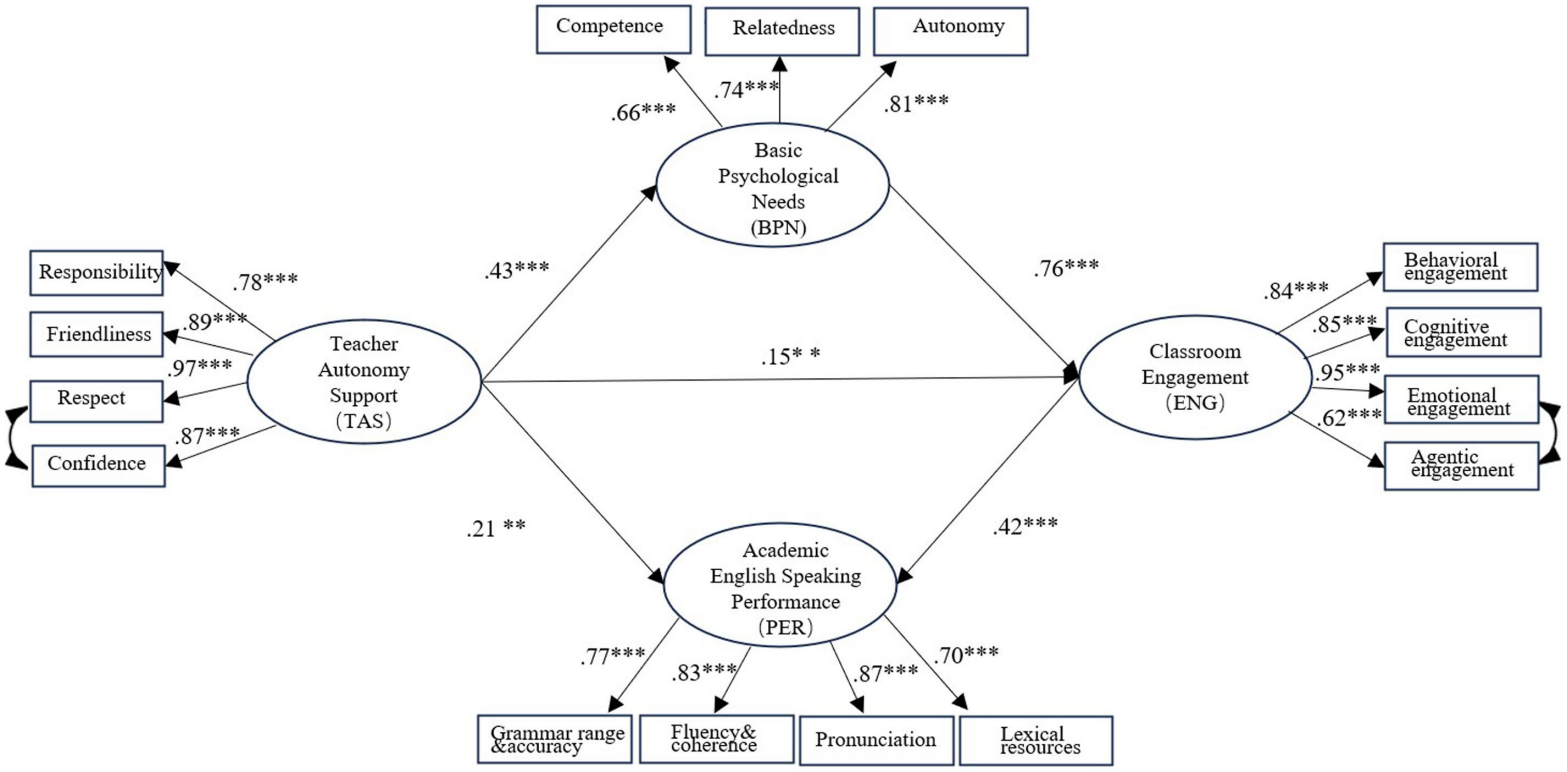
The final model of teacher autonomy support, basic psychological needs, classroom engagement, and academic English speaking performance. *Note:* **p < 0.01. ***p < 0.001.

In the first step, the results showed that the data failed to fit the measurement moderately (*χ*^2^ = 250.39, *df* = 84, *p* < 0.001, CFI = 0.935, TLI = 0.919, RMSEA = 0.09, 90% CI [0.077, 0.103], SRMR = 0.048). According to the model modification report from Mplus software, we modified the model by adding error covariances between “showing respect” and “conveying confidence” as well as between “emotional engagement” and “agentic engagement.” It was reasonable, as these error covariances underlined similar teacher autonomy support and engagement scale dimensions. The final model fit the data well (*χ*^2^ = 180.24, *df* = 82, *p* < 0.001, CFI = 0.962, TLI = 0.951, RMSEA = 0.056, 90% CI [0.056, 0.084], SRMR = 0.046). In the second step, SEM was performed to test the hypothesized SSMMD model, which was a covariance-equivalent model of the CFA model in step 1; the model has the same goodness of fit as the CFA model: *χ*^2^ = 196.07, *df* = 83, *p* < 0.001. The standardized parameter estimates of the hypothesized model are shown in Figure 3. The SEM results showed that teacher autonomy support directly predicted students’ basic psychological needs (*β* = 0.43, *p* < 0.001), classroom engagement (*β* = 0.15, *p* < 0.01), and academic English speaking performance (*β* = 0.21, *p* < 0.01).

In addition, basic psychological needs significantly predicted the students’ engagement (*β* = 0.76, *p* < 0.001), and in turn, engagement was a significant contributor to the speaking performance (*β* = 0.42, *p* < 0.001). According to the indirect effect analysis results (see Table 2), teacher autonomy support indirectly affected students’ English speaking performance through two mediators: basic psychological needs and engagement. There are two indirect paths path 1: TAS → ENG → PER (*β* = 0.063, S. E. = 0.026, *p* = 0.00, 95% BCCI = [0.020, 0.123]); and path 2: TAS → BPN → ENG → PER (*β* = 0.137, S. E. = 0.032, *p* = 0.00, 95% BCCI = [0.083, 0.208]).

**Table 2:** Indirect effects of teacher autonomy support on academic English speaking performance.

	Effect	95% BCCI low	95% BCCI high
TAS-ENG-PER	0.063	0.020	0.123
TAS-BPN-ENG-PER	0.137	0.083	0.208
Total indirect	0.200	0.128	0.292

BCCI, Bootstrap confidence interval.

### 5.3 Interview responses

The follow-up semi-structured interviews were conducted based on the TAS scores results. The detailed information of students (e.g., age, gender, questionnaire scores, and speaking performance) was displayed in Table 3. To provide complementary information for the quantitative findings, we focused primarily on students’ responses concerning the subconstructs of teacher autonomy support (namely, friendliness, responsibility, showing respect, and confidence), various forms of engagement (emotional, agentic, behavioral, and cognitive engagement), and the three elements of BPN (autonomy, relatedness, and competence).

**Table 3:** Information of 12 interviewees.

		Age	Gender	TAS	BPN	ENG	PER
Group 1	Student A	18	Male	3.2	4.0	4.3	4.5
	Student B	17	Male	3.4	4.5	4.0	4.0
	Student C	17	Male	3.3	3.3	3.2	4.0
	Student D	18	Female	3.1	3.8	3.5	4.5
	Student E	18	Female	3.4	4.8	4.5	4.5
	Student F	19	Female	3.0	4.8	4.7	4.5
Group 2	Student G	18	Male	4.6	6.0	6.0	6.0
	Student H	18	Male	4.7	4.9	5.3	5.5
	Student I	18	Male	4.6	4.8	6.0	5.5
	Student J	18	Male	4.8	5.9	6.3	5.5
	Student K	18	Female	4.4	5.2	5.5	5.5
	Student L	18	Female	4.8	5.9	6.3	6.0

While students from the two groups had different levels of perceived teacher autonomy support, they unanimously highlighted the close association between teacher autonomy support, engagement, and speaking performance. The academic English speaking teacher facilitates classroom engagement through various forms of support. For instance, student L (group 2, high-TAS student) noted, “*The teacher always helps us form into collaborative speaking groups and ensures everyone in the group has a role to play in the speaking activities* (responsibility and showing respect). *During the presentation session, the feedback from the teacher was found constructive and supportive, and I was encouraged to be more active in the tasks* (emotional engagement).” Students’ satisfaction with teacher autonomy support and engagement could facilitate academic English speaking performance and vice versa. Some students indicated that teachers’ immediate and encouraging feedback in the classroom (showing respect) empowered them to put more effort (behavioral engagement) into academic speaking. For instance, student J (group 2) said, “*One day, my speaking teacher praised my speaking after I delivered a 3-min academic speech, and she said the speech was clear and logical. You know, it was my academic debut and it was a huge boost to my confidence* (confidence). *Since then, I have kept working hard on academic English speaking* (behavioral engagement). *The improvement is significant*.” In scenarios where students perceive a lack of adequate autonomy support from their instructors, it often results in a correspondingly diminished level of classroom engagement and, thus, a worse speaking result; student B (from the low-TAS group) said, “*I did not think I worked that hard in the classroom. Because my English listening skills are weak, I find it impossible to follow the teachers’ instructions* (confidence). *I had no idea what to do with the task* (cognitive engagement). *My speaking level is even worse this semester*.”

Students from the two groups also mentioned that TAS was related to their BPN in academic English speaking, and those who received greater autonomy support from teachers typically also exhibited higher levels of basic psychological needs satisfaction, such as “*The teacher is familiar with what the young people fancy, and he is a humorous guy. I suppose he is more of my buddy* (friendliness) *and gives me a lot of care in my learning* (relatedness). *I am delighted with his autonomy support, which I believe is very important*” (student K from group 2); “*I am a reserved student, and I feel shy to communicate with my speaking teacher in and out of the classroom* (confidence). *I hope the teacher will inspire my interest in the class* (relatedness) *and help me deal with my problems of poor speaking* (competence)” (student D from group 1).

When asked to elaborate on TAS in academic English class, both groups of students indicated that they believed that teachers’ guidance and assistance were crucial for their basic psychological needs, engagement, and learning performance. High-TAS students explained specifically how their teacher’s instruction of learning resources on new media effectively (responsibility) fascinated them and flexibly helped speed up their improvement in speaking learning. For instance, student I from group 2 said, “*I admire my speaking teacher who has keenly shared in class how to make English videos on social media* (responsibility), *and his help inspired me to improve my speaking learning* via *social media* (autonomy). *Although I started posting videos on Chinese TikTok five years ago, most were dubbed in Chinese. I made several episodes about Chinese marine culture in English this term* (autonomy). *There are 328 fans now, including my speaking teacher, whose experience, techniques, and speaking strategies facilitated my way forward. I spent almost 1 h every day drafting and shooting* (behavioral engagement). *My efforts paid off, and my speaking fluency and accuracy improved a lot*.” Meanwhile, Student D from the low-TAS group noted that she was sometimes absent-minded during speaking classes with the thought that the teacher would not recognize her reluctance to talk (responsibility). Therefore, she did not have any motivation to learn and speak in class (relatedness) and chose to play with the mobile phones and keep silent (behavioral engagement). Her academic English speaking ability got worse and worse. Meanwhile, some low-TAS students attributed their low TAS to their lack of communication with the teacher. Student C said, “*I am from a small village, and my English is not as proficient as my classmates’. I always avoid talking with teachers and students in English* (confidence), *so my classmates are also not that willing to cooperate with me* (relatedness), a*nd that contributed to my lack of interest in and devotion to this course* (emotional engagement). *As a result, my speaking level was not so good as that in my high school*.”

## 6 Discussion

This study investigated the relationships between teacher autonomy support, basic psychological needs, classroom engagement, and academic English speaking performance in an academic English speaking classroom. It was found that students’ perceived teacher support for their learning autonomy can directly predict their final speaking performance; teachers’ autonomy support can also indirectly impact students’ performance via the mediation of basic psychological needs and classroom engagement. Furthermore, participants’ interview responses bolstered the quantitative findings that students who perceived autonomy support from their teachers were more likely to feel more motivated regarding their basic psychological needs in student-teacher relationships and, in turn, engaged more in their speaking learning, consequently achieving higher grades.

### 6.1 The direct effect of teacher autonomy support on basic psychological needs, classroom engagement, and academic English speaking performance

This study confirmed that teacher autonomy support, as a contextual factor, played a significant role in students’ learning (e.g., Skinner et al., 2009; Lietaert et al., 2015; Joe et al., 2017; Wang et al., 2017; Dincer et al., 2019). As previous SDT theorists argued (e.g., Patrick et al., 2011; Skinner and Zimmer-Gembeck, 2011), students who perceived more caring and support for their learning from teachers tended to present increased self-respect, confidence, relatedness, and responsibility for the classroom. In addition, students may also proactively engage in the classroom in the interesting and attractive learning environment created by teachers. As some students stated in this study, teachers actively engaging in the discussion enhanced the student-teacher interaction, encouraging students to contribute more to the class. One student (Student I) noted that the teacher responded to each of the students’ questions, provided constructive feedback on fluency, pronunciation, vocabulary, coherence, and grammar, and never brushed them off; the teacher also offered personalized feedback on after-class assignment; the student was thus motivated to raise his hand and answer questions in class, striving for more opportunities to improve himself. This finding was consistent with Lietaert et al.’s (2015) study concluding that teacher support positively predicted students’ engagement in language classrooms. According to our interview responses, teachers provided immediate and constructive feedback to students’ oral products in the academic English speaking classroom, which motivated students to improve their expression. Students’ interest in academic speaking was thus generated, encouraging them to enjoy expressing themselves in and practicing outside the classroom. However, if students hold negative perceptions of teacher support, such as teachers’ attention and encouragement in the classroom, it might cause their disengagement in communication with peers and teachers. Student B mentioned that the teacher did not focus on him individually, so he could hide in the corner and avoid eye contact with the teacher; that way, he avoided any interactions. Students’ negative perceptions of teacher support may affect their intended effort in L2 communication (Zarrinabadi et al., 2022).

Furthermore, it was noted that students’ perceptions of teacher support directly predicted their speaking performance. This indicated that when students perceived teachers’ patience and strong professional ability, they were more likely to gain success in L2 speaking. Student L said the teacher acknowledged her efforts in each speaking task and gave suggestions, helping her do even better in the next assignment. Her improvement thus accumulated bit by bit. That does not align with the previous argument that teacher support cannot predict students’ L2 learning outcomes (e.g., Dincer et al., 2019). Such discrepancy may be attributed to the different learning contexts. This study primarily focused on EFL learners’ academic speaking in China, which perhaps is more needed for first-year university students in this study. Before entering university, most first-year students often lack substantial opportunities for academic English speaking practice in their text-oriented high-school language classes (Liu, 2006). This transition to higher education marks a significant shift as they encounter the dynamic environment of university academic English-speaking classes. Unlike their previous L2 learning experiences, language teaching becomes distinctly more student-centered, with teachers fostering an autonomous atmosphere where students are encouraged to participate and express themselves actively. Students are more deeply engaged and motivated in such a vibrant and interactive classroom environment. For example, Student H was impressed by a challenging debate task in the academic English speaking class. The intense interaction and eagerness to win effectively activated students’ critical thinking and logical expression in academic English. Aligning with this observation, Liu and Zhang (2023) stated that effective teacher autonomy support in these dynamic academic settings can markedly improve students’ motivation and engagement, subsequently leading to enhanced academic language outcomes. Autonomy-supportive teachers thus appear key to successful language acquisition at the university level.

### 6.2 The indirect effects of teacher autonomy support on academic English speaking performance via BPN and classroom engagement

We also found that teacher autonomy support influenced academic English speaking performance via classroom engagement. A higher level of perceived teacher autonomy support can prompt students’ classroom engagement in the speaking course and achieve satisfactory speaking performance. Teachers offer positive support to students’ learning autonomy, during which students tend to have positive emotional reactions (e.g., enjoyment) in learning (Wang et al., 2017). Student J mentioned that the teacher offered personalized advice and recommended online resources, like YouTube podcasts. His speaking fluency had dramatically improved by mimicking the IELTS logic in the podcasts and actively participating in class. With a high level of positive emotional engagement, students might put more time and energy into speaking and obtain a high speaking grade (Lietaert et al., 2015; Mackey and Gass, 2015; Dincer et al., 2019). In addition to emotional engagement, the information provided by students in the interview also corroborated the pivotal role of teacher autonomy support in students’ behavioral engagement. Teachers usually design class activities such as group discussions, debates, and peer evaluation to establish an interactive and coherent speaking class, which might stimulate more language outputs (see Student H).

Furthermore, teacher autonomy support can influence performance through the mediation of basic psychological needs and classroom engagement in this study. As previous researchers maintained, the more that teachers’ actions and classroom dynamics can satisfy students’ psychological needs, the more that students actively involve themselves in their learning activities, allowing them to learn more and to show higher academic achievement (Ryan and Deci, 2000, 2017; Reeve, 2012; Noels, 2013; Dincer et al., 2019). That is because when basic psychological needs are met, students may have higher motivations to commit to the task toward mastery through active engagement (Reeve, 2012; McEown et al., 2014; Oga-Baldwin et al., 2017). For example, in this study, we discovered that students who perceived their teachers as more supportive of their autonomy had a higher level of satisfaction regarding their autonomy, competence, and sense of connection in academic English speaking learning. When they fail to achieve their short-term goals, they perform remedial actions (e.g., requesting help from teachers) to promote their learning. Consequently, students with better fulfilled psychological needs exhibited more significant levels of engagement across all four dimensions. It was also assumed that due to adequate teacher autonomy support and essential psychological satisfaction, they consider challenges and failures as opportunities to develop their language skills and competence (Lou and Noels, 2017).

Based on interview data, students who felt a lack of autonomy support from their teachers often viewed improving academic English speaking as daunting. They noted the complexities of academic English, including explanation, description, exemplification, and counter-argumentation. They perceived low teacher autonomy support, and their academic English speaking ability saw no progress. Cultural and educational backgrounds and personal experiences partially influenced their perceptions. For instance, one student (Student G) remained silent during a lesson on counter-argumentation, feeling it was culturally inappropriate to challenge teachers, reflecting deep-rooted respect for teacher authority in Chinese culture. This example highlights students’ diverse challenges of adapting to academic English speaking within oriental cultural contexts. Consequently, such negative self-perception undermined their classroom engagement and academic speaking ability (Ryan and Deci, 2000). Therefore, it is important to understand the balance between maintaining cultural reverence for teachers and fostering an environment where students feel empowered to take initiative in their learning.

## 7 Conclusion

This study has bridged a significant gap in understanding the relationships between teacher autonomy support, basic psychological needs, classroom engagement, and speaking performance in academic English speaking classrooms. It reinforces the idea that teacher autonomy support is not just about academic freedom but also involves fostering an engaging and motivating learning environment. Our findings underscore the notable direct effect of teacher autonomy support on academic English speaking performance, as well as two key indirect routes.

Firstly, the notable impact of teacher autonomy support on speaking performance necessitates the adoption of strategies that enhance autonomy in academic English learning. Strategies such as aligning academic content with student interests, offering assignment choices, promoting student-led projects, and providing personalized feedback can significantly enhance L2-speaking teachers’ autonomy support (Rahimi and Wee Ong, 2023). Moreover, teachers should improve their sense of responsibility and show respect and friendliness, which can significantly boost students’ confidence and encourage them to make an intended effort in the learning process.

Secondly, student-centered teaching in L2 speaking classes is further substantiated by this research. This approach often incorporates elements like collaborative tasks, peer tutoring, and the creation of a non-judgmental environment, enabling students to express their opinions freely, for instance, through class message forums (Solhi, 2023). This approach is especially pertinent for introverted students who may benefit significantly from a greater degree of teacher autonomy support (Saroughi and Cheema, 2022).

Thirdly, within the cultural framework of Chinese Confucianism, language teachers in China must be highly aware of the significant impact that culture has on students’ initiative and openness in speaking English in the academic context. Teachers could effectively integrate motivation and engagement interventions with speaking strategy instruction in their classrooms. They also need to establish meaningful connections with the students and create a caring and inclusive learning environment, which helps provide ongoing and relevant autonomy support tailored to the student’s basic psychological needs and fosters a more proactive and open classroom climate. That may build up students’ willingness to speak, which is an essential factor for L2 speaking success (Zarrinabadi et al., 2022). For example, attentive listening and expressing interest in students’ remarks can enhance their motivation to communicate in speaking tasks. This approach can serve as an effective icebreaker; moreover, assigning online speaking projects via social media that promote collaborative learning appears to be a promising approach to grappling with such cultural influence (Wang, 2015; Solhi, 2023).

We also acknowledge that there are several limitations to this study. The primary limitation is the cross-sectional nature of the research design, which cannot demonstrate the causal effect of teacher support and other individual factors. Therefore, longitudinal studies can be considered in future research. The second limitation might be that male students in this study outnumbered their female counterparts, which may bias our interpretation of the findings. However, the imbalanced distribution of gender is commonly seen among engineering major students. Future investigations could extend our study to a broader population. Third, although this study provides a general understanding of the relationships among teacher autonomy support, basic psychological needs, engagement, and L2 speaking, the specific relations between the elements of these variables still need further empirical studies.

## Data availability statement

The raw data supporting the conclusions of this article will be made available by the authors, without undue reservation.

## Ethics statement

The studies involving humans were approved by the Academic Committee of School of Foreign Languages of Dalian Maritime University. The studies were conducted in accordance with the local legislation and institutional requirements. The participants provided their written informed consent to participate in this study.

## Author contributions

YW: Conceptualization, Data curation, Funding acquisition, Investigation, Project administration, Writing – original draft. WL: Writing – review & editing. XL: Conceptualization, Resources, Validation, Writing – review & editing. PZ: Conceptualization, Methodology, Supervision, Validation, Writing – original draft, Writing – review & editing.

## Footnotes

^1^ https://www.wjx.cn/
