# Supplementary material for: Exploring the effect of teacher autonomy support on Chinese EFL undergraduates’ academic English speaking performance through the mediation of basic psychological needs and classroom engagement
Source: Front Psychol. 2024 Feb 20;15:1323713. doi: 10.3389/fpsyg.2024.1323713 (PMC10913198; doi:10.3389/fpsyg.2024.1323713)
Supplement: Supplementary file 1 [file Table_1.docx]

**Questionaries**

本问卷旨在搜集您作为大学生对于学术英语口语课堂学习的看法或感受。答案没有对错，以帮助学术口语教师有效改进学术口语课程的教学全过程。请您认真阅读每一条目后做出准确的选择。本问卷仅用于研究用途，相关数据不会告知任课教师，不会传播给其他个人或组织。非常感谢您的合作，祝生活愉快，一切顺利！

第一部分是学生基本信息，本研究将对本部分出现的姓名严格保密。

姓名 [填空题] *

请填写汉字

_________________________________

性别 [单选题] *

| ○男 | ○女 |
| --- | --- |

专业 [单选题] *

| ○土木 | ○电子 | ○机械 |
| --- | --- | --- |

高考英语成绩（满分150） [填空题] *

_________________________________

多邻国成绩（最近一次出分） [填空题] *

_________________________________

第二部分是学生学术口语学习的基本心理需求调查，涉及学习的自主性、学习能力和相关性。其中，“1”代表“非常不同意”；“2”代表“不同意”；“3”代表“比较不同意”；“4”代表“中立”；“5”代表“比较同意”；“6”代表“同意”；“7”代表“非常同意”。请结合自身，表明您在多大程度上同意或不同意该条目的陈述，并在对应的选项上打勾。

1.我可以自由决定自己的学术口语学习进度。 [单选题] *

| ○1.非常不同意 | ○2.不同意 | ○3.比较不同意 | ○4.中立 | ○5.比较同意 | ○6.同意 | ○7.非常同意 |
| --- | --- | --- | --- | --- | --- | --- |

BPN-Auto2

2.在学习学术口语时，我可以自己选择要做的学习任务。 [单选题] *

| ○1.非常不同意 | ○2.不同意 | ○3.比较不同意 | ○4.中立 | ○5.比较同意 | ○6.同意 | ○7.非常同意 |
| --- | --- | --- | --- | --- | --- | --- |

BPN-Auto3

3.我的学术口语老师允许同学自由选择学术口语的学习方法。 [单选题] *

| ○1.非常不同意 | ○2.不同意 | ○3.比较不同意 | ○4.中立 | ○5.比较同意 | ○6.同意 | ○7.非常同意 |
| --- | --- | --- | --- | --- | --- | --- |

4.在学术口语课堂中，我的老师允许我自主进行学术口语练习。 [单选题] *

| ○1.非常不同意 | ○2.不同意 | ○3.比较不同意 | ○4.中立 | ○5.比较同意 | ○6.同意 | ○7.非常同意 |
| --- | --- | --- | --- | --- | --- | --- |

5.我觉得我有能力学习学术口语。 [单选题] *

| ○1.非常不同意 | ○2.不同意 | ○3.比较不同意 | ○4.中立 | ○5.比较同意 | ○6.同意 | ○7.非常同意 |
| --- | --- | --- | --- | --- | --- | --- |

BPN-Com2

6.我可以成为一个成功的学术口语学习者。 [单选题] *

| ○1.非常不同意 | ○2.不同意 | ○3.比较不同意 | ○4.中立 | ○5.比较同意 | ○6.同意 | ○7.非常同意 |
| --- | --- | --- | --- | --- | --- | --- |

7.我有足够的能力去迎接学术口语学习中的挑战和任务。 [单选题] *

| ○1.非常不同意 | ○2.不同意 | ○3.比较不同意 | ○4.中立 | ○5.比较同意 | ○6.同意 | ○7.非常同意 |
| --- | --- | --- | --- | --- | --- | --- |

8.我在学术口语课上很有成就感。 [单选题] *

| ○1.非常不同意 | ○2.不同意 | ○3.比较不同意 | ○4.中立 | ○5.比较同意 | ○6.同意 | ○7.非常同意 |
| --- | --- | --- | --- | --- | --- | --- |

9.我的学术口语老师对我很友好和亲切。 [单选题] *

| ○1.非常不同意 | ○2.不同意 | ○3.比较不同意 | ○4.中立 | ○5.比较同意 | ○6.同意 | ○7.非常同意 |
| --- | --- | --- | --- | --- | --- | --- |

10.我的学术口语老师能够体会理解学生口语学习中的问题。 [单选题] *

| ○1.非常不同意 | ○2.不同意 | ○3.比较不同意 | ○4.中立 | ○5.比较同意 | ○6.同意 | ○7.非常同意 |
| --- | --- | --- | --- | --- | --- | --- |

11.我的同学在学习学术口语的过程中愿意帮助我，与我合作。 [单选题] *

| ○1.非常不同意 | ○2.不同意 | ○3.比较不同意 | ○4.中立 | ○5.比较同意 | ○6.同意 | ○7.非常同意 |
| --- | --- | --- | --- | --- | --- | --- |

12.我的学术口语老师关心我的进步。 [单选题] *

| ○1.非常不同意 | ○2.不同意 | ○3.比较不同意 | ○4.中立 | ○5.比较同意 | ○6.同意 | ○7.非常同意 |
| --- | --- | --- | --- | --- | --- | --- |

第三部分是学术口语学习投入调查，将从情感投入、行为投入、认知投入和能动性投入四个维度展开。其中，“1”代表“非常不同意”；“2”代表“不同意”；“3”代表“比较不同意”；“4”代表“中立”；“5”代表“比较同意”；“6”代表“同意”；“7”代表“非常同意”。请结合自身，表明您在多大程度上同意或不同意该条目的陈述，并在对应的选项上打勾。

13.我会在学术口语课上主动提问。 [单选题] *

| ○1.非常不同意 | ○2.不同意 | ○3.比较不同意 | ○4.中立 | ○5.比较同意 | ○6.同意 | ○7.非常同意 |
| --- | --- | --- | --- | --- | --- | --- |

14.我会告诉老师我喜欢什么或不喜欢什么。 [单选题] *

| ○1.非常不同意 | ○2.不同意 | ○3.比较不同意 | ○4.中立 | ○5.比较同意 | ○6.同意 | ○7.非常同意 |
| --- | --- | --- | --- | --- | --- | --- |

15.我会让学术口语老师知道我对什么内容感兴趣。 [单选题] *

| ○1.非常不同意 | ○2.不同意 | ○3.比较不同意 | ○4.中立 | ○5.比较同意 | ○6.同意 | ○7.非常同意 |
| --- | --- | --- | --- | --- | --- | --- |

16.在学术口语的课堂上，我会表达我的喜好和意见。 [单选题] *

| ○1.非常不同意 | ○2.不同意 | ○3.比较不同意 | ○4.中立 | ○5.比较同意 | ○6.同意 | ○7.非常同意 |
| --- | --- | --- | --- | --- | --- | --- |

17.在学术口语的课堂上，我提出了一些关于如何让课堂更好的建议。 [单选题] *

| ○1.非常不同意 | ○2.不同意 | ○3.比较不同意 | ○4.中立 | ○5.比较同意 | ○6.同意 | ○7.非常同意 |
| --- | --- | --- | --- | --- | --- | --- |

18.在学术口语的课堂上，我会认真听讲。 [单选题] *

| ○1.非常不同意 | ○2.不同意 | ○3.比较不同意 | ○4.中立 | ○5.比较同意 | ○6.同意 | ○7.非常同意 |
| --- | --- | --- | --- | --- | --- | --- |

19.我在学术口语学习中非常努力。 [单选题] *

| ○1.非常不同意 | ○2.不同意 | ○3.比较不同意 | ○4.中立 | ○5.比较同意 | ○6.同意 | ○7.非常同意 |
| --- | --- | --- | --- | --- | --- | --- |

20.在学术口语课堂上，老师每次讲解新的话题，我都会认真聆听。 [单选题] *

| ○1.非常不同意 | ○2.不同意 | ○3.比较不同意 | ○4.中立 | ○5.比较同意 | ○6.同意 | ○7.非常同意 |
| --- | --- | --- | --- | --- | --- | --- |

21.在学术口语课堂上，老师每次讲解新的知识，我都会努力学习并理解。 [单选题] *

| ○1.非常不同意 | ○2.不同意 | ○3.比较不同意 | ○4.中立 | ○5.比较同意 | ○6.同意 | ○7.非常同意 |
| --- | --- | --- | --- | --- | --- | --- |

22.我在学术口语课堂上注意力集中。 [单选题] *

| ○1.非常不同意 | ○2.不同意 | ○3.比较不同意 | ○4.中立 | ○5.比较同意 | ○6.同意 | ○7.非常同意 |
| --- | --- | --- | --- | --- | --- | --- |

23.我喜欢在学术口语课堂上学习新知识。 [单选题] *

| ○1.非常不同意 | ○2.不同意 | ○3.比较不同意 | ○4.中立 | ○5.比较同意 | ○6.同意 | ○7.非常同意 |
| --- | --- | --- | --- | --- | --- | --- |

24.在学术口语课堂中，我对学习任务饶有兴趣。 [单选题] *

| ○1.非常不同意 | ○2.不同意 | ○3.比较不同意 | ○4.中立 | ○5.比较同意 | ○6.同意 | ○7.非常同意 |
| --- | --- | --- | --- | --- | --- | --- |

25.在学术口语课堂，我对学习内容充满好奇。 [单选题] *

| ○1.非常不同意 | ○2.不同意 | ○3.比较不同意 | ○4.中立 | ○5.比较同意 | ○6.同意 | ○7.非常同意 |
| --- | --- | --- | --- | --- | --- | --- |

26.在学术口语课上，我心情愉悦。 [单选题] *

| ○1.非常不同意 | ○2.不同意 | ○3.比较不同意 | ○4.中立 | ○5.比较同意 | ○6.同意 | ○7.非常同意 |
| --- | --- | --- | --- | --- | --- | --- |

27.完成学术口语作业的时候，我尝试把我正在学的知识和我的已知联系起来。 [单选题] *

| ○1.非常不同意 | ○2.不同意 | ○3.比较不同意 | ○4.中立 | ○5.比较同意 | ○6.同意 | ○7.非常同意 |
| --- | --- | --- | --- | --- | --- | --- |

28.学习学术口语的时候，我试着把我正在学的知识和我的经历联系起来。 [单选题] *

| ○1.非常不同意 | ○2.不同意 | ○3.比较不同意 | ○4.中立 | ○5.比较同意 | ○6.同意 | ○7.非常同意 |
| --- | --- | --- | --- | --- | --- | --- |

29.学习学术口语的时候，我会努力整合知识，直至学会当下的内容。 [单选题] *

| ○1.非常不同意 | ○2.不同意 | ○3.比较不同意 | ○4.中立 | ○5.比较同意 | ○6.同意 | ○7.非常同意 |
| --- | --- | --- | --- | --- | --- | --- |

30.学习学术口语的时候，对于所学的重要知识点，我会通过举例的方法帮助理解。 [单选题] *

| ○1.非常不同意 | ○2.不同意 | ○3.比较不同意 | ○4.中立 | ○5.比较同意 | ○6.同意 | ○7.非常同意 |
| --- | --- | --- | --- | --- | --- | --- |

31.每次学习学术口语前，我会制定学习目标。 [单选题] *

| ○1.非常不同意 | ○2.不同意 | ○3.比较不同意 | ○4.中立 | ○5.比较同意 | ○6.同意 | ○7.非常同意 |
| --- | --- | --- | --- | --- | --- | --- |

32.完成学术口语的作业时，我都会留时间进行复习，回顾所学知识。 [单选题] *

| ○1.非常不同意 | ○2.不同意 | ○3.比较不同意 | ○4.中立 | ○5.比较同意 | ○6.同意 | ○7.非常同意 |
| --- | --- | --- | --- | --- | --- | --- |

33.在学习学术口语时，我会就相关问题明确自己理解的程度，而不仅仅关注我是否得到了正确的答案。 [单选题] *

| ○1.非常不同意 | ○2.不同意 | ○3.比较不同意 | ○4.中立 | ○5.比较同意 | ○6.同意 | ○7.非常同意 |
| --- | --- | --- | --- | --- | --- | --- |

34.在学习学术口语时，如果相关题目难以理解，我会试图改变学习和理解的方式。 [单选题] *

| ○1.非常不同意 | ○2.不同意 | ○3.比较不同意 | ○4.中立 | ○5.比较同意 | ○6.同意 | ○7.非常同意 |
| --- | --- | --- | --- | --- | --- | --- |

第四部分为教师自主性学习支持调查，包含责任心、亲切度、尊重和自信心支持四个维度。其中，“1”代表“从不”；“2”代表“不频繁”；“3”代表“比较频繁”；“4”代表“频繁”；“5”代表“非常频繁”；请结合自身感受，体会教师自主性支持相关事件的发生频率，并在对应的选项上打勾35.我的学术口语老师让我们杜绝学习懈怠和拖延行为。

[单选题] *

| ○1.从不 | ○2.不频繁 | ○3.比较频繁 | ○4.频繁 | ○5.非常频繁 |  |  |
| --- | --- | --- | --- | --- | --- | --- |

36.我的学术口语老师对成绩不佳的同学给予额外关注。 [单选题] *

| ○1.从不 | ○2.不频繁 | ○3.比较频繁 | ○4.频繁 | ○5.非常频繁 |  |  |
| --- | --- | --- | --- | --- | --- | --- |

37.我的学术口语老师会专注于教学辅助手段的应用和教学活动的开展 [单选题] *

| ○1.从不 | ○2.不频繁 | ○3.比较频繁 | ○4.频繁 | ○5.非常频繁 |  |  |
| --- | --- | --- | --- | --- | --- | --- |

38.我的学术口语老师会负责任地解决学生的问题。 [单选题] *

| ○1.从不 | ○2.不频繁 | ○3.比较频繁 | ○4.频繁 | ○5.非常频繁 |  |  |
| --- | --- | --- | --- | --- | --- | --- |

39.我的学术口语老师会及时给予课堂表现反馈。 [单选题] *

| ○1.从不 | ○2.不频繁 | ○3.比较频繁 | ○4.频繁 | ○5.非常频繁 |  |  |
| --- | --- | --- | --- | --- | --- | --- |

40.我的学术口语老师脾气不急不躁。 [单选题] *

| ○1.从不 | ○2.不频繁 | ○3.比较频繁 | ○4.频繁 | ○5.非常频繁 |  |  |
| --- | --- | --- | --- | --- | --- | --- |

41.我的学术口语老师有幽默感。 [单选题] *

| ○1.从不 | ○2.不频繁 | ○3.比较频繁 | ○4.频繁 | ○5.非常频繁 |  |  |
| --- | --- | --- | --- | --- | --- | --- |

42.我的学术口语老师愿意倾听我的问题。 [单选题] *

| ○1.从不 | ○2.不频繁 | ○3.比较频繁 | ○4.频繁 | ○5.非常频繁 |  |  |
| --- | --- | --- | --- | --- | --- | --- |

43.我的学术口语老师了解学生的弱点。 [单选题] *

| ○1.从不 | ○2.不频繁 | ○3.比较频繁 | ○4.频繁 | ○5.非常频繁 |  |  |
| --- | --- | --- | --- | --- | --- | --- |

44.我的学术口语老师即使在课堂外也会平易近人。 [单选题] *

| ○1.从不 | ○2.不频繁 | ○3.比较频繁 | ○4.频繁 | ○5.非常频繁 |  |  |
| --- | --- | --- | --- | --- | --- | --- |

45.我的学术口语老师鼓励我自主选用学习方法。 [单选题] *

| ○1.从不 | ○2.不频繁 | ○3.比较频繁 | ○4.频繁 | ○5.非常频繁 |  |  |
| --- | --- | --- | --- | --- | --- | --- |

46.我的学术口语老师和学生交谈时语气温和。 [单选题] *

| ○1.从不 | ○2.不频繁 | ○3.比较频繁 | ○4.频繁 | ○5.非常频繁 |  |  |
| --- | --- | --- | --- | --- | --- | --- |

47.我的学术口语老师会欣赏我的优点和学习成果。 [单选题] *

| ○1.从不 | ○2.不频繁 | ○3.比较频繁 | ○4.频繁 | ○5.非常频繁 |  |  |
| --- | --- | --- | --- | --- | --- | --- |

48.我的学术口语老师会尊重学生。 [单选题] *

| ○1.从不 | ○2.不频繁 | ○3.比较频繁 | ○4.频繁 | ○5.非常频繁 |  |  |
| --- | --- | --- | --- | --- | --- | --- |

49.我的学术口语老师对学生一视同仁。 [单选题] *

| ○1.从不 | ○2.不频繁 | ○3.比较频繁 | ○4.频繁 | ○5.非常频繁 |  |  |
| --- | --- | --- | --- | --- | --- | --- |

50.我的学术口语老师会布置跟学习内容相关的任务和作业。 [单选题] *

| ○1.从不 | ○2.不频繁 | ○3.比较频繁 | ○4.频繁 | ○5.非常频繁 |  |  |
| --- | --- | --- | --- | --- | --- | --- |

51.我的学术口语老师会组织答疑环节。 [单选题] *

| ○1.从不 | ○2.不频繁 | ○3.比较频繁 | ○4.频繁 | ○5.非常频繁 |  |  |
| --- | --- | --- | --- | --- | --- | --- |

52.我的学术口语老师能够对学生的错误持包容心态。 [单选题] *

| ○1.从不 | ○2.不频繁 | ○3.比较频繁 | ○4.频繁 | ○5.非常频繁 |  |  |
| --- | --- | --- | --- | --- | --- | --- |

53.我的学术口语老师不会吝啬赞美之词。 [单选题] *

| ○1.从不 | ○2.不频繁 | ○3.比较频繁 | ○4.频繁 | ○5.非常频繁 |  |  |
| --- | --- | --- | --- | --- | --- | --- |

54.我的学术口语老师鼓励学生设定高标准的学习目标。 [单选题] *

| ○1.从不 | ○2.不频繁 | ○3.比较频繁 | ○4.频繁 | ○5.非常频繁 |  |  |
| --- | --- | --- | --- | --- | --- | --- |
