# Supplementary material for: Exploring the effect of teacher autonomy support on Chinese EFL undergraduates’ academic English speaking performance through the mediation of basic psychological needs and classroom engagement
Source: Front Psychol. 2024 Feb 20;15:1323713. doi: 10.3389/fpsyg.2024.1323713 (PMC10913198; doi:10.3389/fpsyg.2024.1323713)
Supplement: Supplementary file 2 [file Table_2.docx]

**Questionaries**

Thank you for considering participation in our survey. This questionnaire seeks to gather insights from you, as a university student, about your experiences and perceptions related to learning spoken English in an academic context. Please know that this survey does not seek "right" or "wrong" answers. Instead, its goal is to collect valuable feedback that can help academic speaking instructors enhance the effectiveness of the teaching methodologies applied in academic speaking courses. Be assured that your responses will be kept confidential and used exclusively for research purposes to improve the academic speaking curriculum. Your participation in this survey is entirely voluntary, and you have the freedom to withdraw at any time without any adverse effects. We value your input greatly and are grateful for your time and cooperation. We wish you all the best in your academic pursuits and a fulfilling life ahead.

**Part 1: Basic Student Information** (All names will be kept confidential in this research.)

Name [Fill in the blank] *

请填写汉字

_________________________________

Gender [Fill in the blank] *

| ○Male | ○Female |
| --- | --- |

Major [Multiple choice] *

| ○Civil Engineering | ○Electrical Engineering | ○Mechanical Engineering |
| --- | --- | --- |

College Entrance Exam English Score (out of 150) [Fill in the blank] *

_________________________________

Duolingo Score (Most recent) [Fill in the blank] *

_________________________________

**Part 2: Basic Psychological Needs Survey for Academic Spoken English Learning**, involving autonomy, competence, and relatedness. The scale ranges from "1" representing "Strongly disagree" to "7" representing "strongly agree". Please indicate the extent to which you agree or disagree with each statement and tick the corresponding option.

1. I am able to freely decide my own pace in academic English speaking learning. [Multiple choice] *

| ○1.Strongly disagree | ○2.Disagree | ○3.Somewhat disagree | ○4.Neutral | ○5.Somewhat agree | ○6.Agree | ○7.Strongly agree |
| --- | --- | --- | --- | --- | --- | --- |

2When learning academic English , I am able to freely choose the learning tasks to do by myself. [Multiple choice] *

| ○1.Strongly disagree | ○2.Disagree | ○3.Somewhat disagree | ○4.Neutral | ○5.Somewhat agree | ○6.Agree | ○7.Strongly agree |
| --- | --- | --- | --- | --- | --- | --- |

3. My academic spoken English teacher allows students to freely choose how we approach academic English speaking learning. [Multiple choice] *

| ○1.Strongly disagree | ○2.Disagree | ○3.Somewhat disagree | ○4.Neutral | ○5.Somewhat agree | ○6.Agree | ○7.Strongly agree |
| --- | --- | --- | --- | --- | --- | --- |

4. My English teacher lets freely practice speaking academic English in the classroom. [Multiple choice] *

| ○1.Strongly disagree | ○2.Disagree | ○3.Somewhat disagree | ○4.Neutral | ○5.Somewhat agree | ○6.Agree | ○7.Strongly agree |
| --- | --- | --- | --- | --- | --- | --- |

5.I feel I am capable of learning academic English speaking. [Multiple choice] *

| ○1.Strongly disagree | ○2.Disagree | ○3.Somewhat disagree | ○4.Neutral | ○5.Somewhat agree | ○6.Agree | ○7.Strongly agree |
| --- | --- | --- | --- | --- | --- | --- |

6.I can be a successful learner of academic English speaking. [Multiple choice] *

| ○1.Strongly disagree | ○2.Disagree | ○3.Somewhat disagree | ○4.Neutral | ○5.Somewhat agree | ○6.Agree | ○7.Strongly agree |
| --- | --- | --- | --- | --- | --- | --- |

7.I am competent enough to meet the challenges and tasks posed in academic English speaking learning. [Multiple choice] *

| ○1.Strongly disagree | ○2.Disagree | ○3.Somewhat disagree | ○4.Neutral | ○5.Somewhat agree | ○6.Agree | ○7.Strongly agree |
| --- | --- | --- | --- | --- | --- | --- |

8.I feel a sense of accomplishment in my academic English speaking classes. [Multiple choice] *

| ○1.Strongly disagree | ○2.Disagree | ○3.Somewhat disagree | ○4.Neutral | ○5.Somewhat agree | ○6.Agree | ○7.Strongly agree |
| --- | --- | --- | --- | --- | --- | --- |

9.My academic spoken English teacher is friendly and cordial with me. [Multiple choice] *

| ○1.Strongly disagree | ○2.Disagree | ○3.Somewhat disagree | ○4.Neutral | ○5.Somewhat agree | ○6.Agree | ○7.Strongly agree |
| --- | --- | --- | --- | --- | --- | --- |

10. My academic spoken English teacher is very understanding about my learning problems in this course. [Multiple choice] *

| ○1.Strongly disagree | ○2.Disagree | ○3.Somewhat disagree | ○4.Neutral | ○5.Somewhat agree | ○6.Agree | ○7.Strongly agree |
| --- | --- | --- | --- | --- | --- | --- |

11.My classmates are willing to help and cooperate with me in the learning process. [Multiple choice] *

| ○1.Strongly disagree | ○2.Disagree | ○3.Somewhat disagree | ○4.Neutral | ○5.Somewhat agree | ○6.Agree | ○7.Strongly agree |
| --- | --- | --- | --- | --- | --- | --- |

12. My academic spoken English teacher cares about my progress. [Multiple choice] *

| ○1.Strongly disagree | ○2.Disagree | ○3.Somewhat disagree | ○4.Neutral | ○5.Somewhat agree | ○6.Agree | ○7.Strongly agree |
| --- | --- | --- | --- | --- | --- | --- |

**Part Three is a survey on academic spoken English engagement,** which will be conducted across four dimensions: emotional engagement, behavioral engagement, cognitive engagement, and agentic engagement. '1' represents 'Strongly disagree'; '2' represents 'Disagree'; '3' represents 'Somewhat disagree'; '4' represents 'Neutral'; '5' represents 'Somewhat agree'; '6' represents 'Agree'; '7' represents 'Strongly agree'. Please indicate the extent to which you agree or disagree with each statement based on your own experience, and mark your choice accordingly.

13. During class, I ask questions. [Multiple choice] *

| ○1.Strongly disagree | ○2.Disagree | ○3.Somewhat disagree | ○4.Neutral | ○5.Somewhat agree | ○6.Agree | ○7.Strongly agree |
| --- | --- | --- | --- | --- | --- | --- |

14. I tell the teacher of academic English speaking what I like and what I dislike. [Multiple choice] *

| ○1.Strongly disagree | ○2.Disagree | ○3.Somewhat disagree | ○4.Neutral | ○5.Somewhat agree | ○6.Agree | ○7.Strongly agree |
| --- | --- | --- | --- | --- | --- | --- |

15.I let my teacher know what I’m interested. [Multiple choice] *

| ○1.Strongly disagree | ○2.Disagree | ○3.Somewhat disagree | ○4.Neutral | ○5.Somewhat agree | ○6.Agree | ○7.Strongly agree |
| --- | --- | --- | --- | --- | --- | --- |

16.During class, I express my preferences and opinions. [Multiple choice] *

| ○1.Strongly disagree | ○2.Disagree | ○3.Somewhat disagree | ○4.Neutral | ○5.Somewhat agree | ○6.Agree | ○7.Strongly agree |
| --- | --- | --- | --- | --- | --- | --- |

17. I offer suggestions about how to make the class of academic English speaking better. [Multiple choice] *

| ○1.Strongly disagree | ○2.Disagree | ○3.Somewhat disagree | ○4.Neutral | ○5.Somewhat agree | ○6.Agree | ○7.Strongly agree |
| --- | --- | --- | --- | --- | --- | --- |

18.I listen carefully in the class of academic English speaking. [Multiple choice] *

| ○1.Strongly disagree | ○2.Disagree | ○3.Somewhat disagree | ○4.Neutral | ○5.Somewhat agree | ○6.Agree | ○7.Strongly agree |
| --- | --- | --- | --- | --- | --- | --- |

19. I try very hard in the course of academic English speaking. [Multiple choice] *

| ○1.Strongly disagree | ○2.Disagree | ○3.Somewhat disagree | ○4.Neutral | ○5.Somewhat agree | ○6.Agree | ○7.Strongly agree |
| --- | --- | --- | --- | --- | --- | --- |

20. The first time my academic spoken English teacher talks about a new topic, I listen very carefully [Multiple choice] *

| ○1.Strongly disagree | ○2.Disagree | ○3.Somewhat disagree | ○4.Neutral | ○5.Somewhat agree | ○6.Agree | ○7.Strongly agree |
| --- | --- | --- | --- | --- | --- | --- |

21. I work hard when we start something new in class. [Multiple choice] *

| ○1.Strongly disagree | ○2.Disagree | ○3.Somewhat disagree | ○4.Neutral | ○5.Somewhat agree | ○6.Agree | ○7.Strongly agree |
| --- | --- | --- | --- | --- | --- | --- |

22. I pay attention in the class of academic English speaking. [Multiple choice] *

| ○1.Strongly disagree | ○2.Disagree | ○3.Somewhat disagree | ○4.Neutral | ○5.Somewhat agree | ○6.Agree | ○7.Strongly agree |
| --- | --- | --- | --- | --- | --- | --- |

23.I enjoy learning new things in the class of academic English speaking. [Multiple choice] *

| ○1.Strongly disagree | ○2.Disagree | ○3.Somewhat disagree | ○4.Neutral | ○5.Somewhat agree | ○6.Agree | ○7.Strongly agree |
| --- | --- | --- | --- | --- | --- | --- |

24.When we work on something in class, I feel interested. [Multiple choice] *

| ○1.Strongly disagree | ○2.Disagree | ○3.Somewhat disagree | ○4.Neutral | ○5.Somewhat agree | ○6.Agree | ○7.Strongly agree |
| --- | --- | --- | --- | --- | --- | --- |

25. When I am in the class of academic English speaking, I feel curious about what we are learning. [Multiple choice] *

| ○1.Strongly disagree | ○2.Disagree | ○3.Somewhat disagree | ○4.Neutral | ○5.Somewhat agree | ○6.Agree | ○7.Strongly agree |
| --- | --- | --- | --- | --- | --- | --- |

26. The class of academic English speaking is fun. [Multiple choice] *

| ○1.Strongly disagree | ○2.Disagree | ○3.Somewhat disagree | ○4.Neutral | ○5.Somewhat agree | ○6.Agree | ○7.Strongly agree |
| --- | --- | --- | --- | --- | --- | --- |

27. When doing the schoolwork of academic English speaking, I try to relate what I’m learning to what I have learnt. [Multiple choice] *

| ○1.Strongly disagree | ○2.Disagree | ○3.Somewhat disagree | ○4.Neutral | ○5.Somewhat agree | ○6.Agree | ○7.Strongly agree |
| --- | --- | --- | --- | --- | --- | --- |

28.When I study academic English speaking, I try to connect what I’m learning with my own experience. [Multiple choice] *

| ○1.Strongly disagree | ○2.Disagree | ○3.Somewhat disagree | ○4.Neutral | ○5.Somewhat agree | ○6.Agree | ○7.Strongly agree |
| --- | --- | --- | --- | --- | --- | --- |

29. I try to make all the different ideas fit together and make sense when I study academic English speaking. [Multiple choice] *

| ○1.Strongly disagree | ○2.Disagree | ○3.Somewhat disagree | ○4.Neutral | ○5.Somewhat agree | ○6.Agree | ○7.Strongly agree |
| --- | --- | --- | --- | --- | --- | --- |

30. I make up my own examples to help me understand the important concepts I study in the academic English speaking courses.[Multiple choice] *

| ○1.Strongly disagree | ○2.Disagree | ○3.Somewhat disagree | ○4.Neutral | ○5.Somewhat agree | ○6.Agree | ○7.Strongly agree |
| --- | --- | --- | --- | --- | --- | --- |

31. Before I begin to study academic English speaking, I think about what I want to get done. [Multiple choice] *

| ○1.Strongly disagree | ○2.Disagree | ○3.Somewhat disagree | ○4.Neutral | ○5.Somewhat agree | ○6.Agree | ○7.Strongly agree |
| --- | --- | --- | --- | --- | --- | --- |

32. When I am working on my schoolwork, I stop once in a while and go over what I have been doing. [Multiple choice] *

| ○1.Strongly disagree | ○2.Disagree | ○3.Somewhat disagree | ○4.Neutral | ○5.Somewhat agree | ○6.Agree | ○7.Strongly agree |
| --- | --- | --- | --- | --- | --- | --- |

33. As I study, I keep track of how much I understand, not just if I am getting the right answers. [Multiple choice] *

| ○1.Strongly disagree | ○2.Disagree | ○3.Somewhat disagree | ○4.Neutral | ○5.Somewhat agree | ○6.Agree | ○7.Strongly agree |
| --- | --- | --- | --- | --- | --- | --- |

34. If what I am working on is difficult to understand, I change the way I learn the material. [Multiple choice] *

| ○1.Strongly disagree | ○2.Disagree | ○3.Somewhat disagree | ○4.Neutral | ○5.Somewhat agree | ○6.Agree | ○7.Strongly agree |
| --- | --- | --- | --- | --- | --- | --- |

**Part Four focuses on the survey of teacher autonomy support**, covering four dimensions: responsibility, friendliness, respect, and confidence support. '1' represents 'Never'; '2' represents 'Rarely'; '3' represents 'Somewhat often'; '4' represents 'Often'; '5' represents 'Very often'. Please reflect on your own feelings and experiences regarding the frequency of teacher autonomy support-related events, and mark the corresponding option accordingly.

35.My academic spoken English teacher discourages tardiness. [Multiple choice] *

| ○1.Never | ○2.Rarely | ○3.Somewhat often | ○4.Often | ○5.Very often |  |  |
| --- | --- | --- | --- | --- | --- | --- |

36.My academic spoken English teacher gives extra attention for weak students.[Multiple choice] *

| ○1.Never | ○2.Rarely | ○3.Somewhat often | ○4.Often | ○5.Very often |  |  |
| --- | --- | --- | --- | --- | --- | --- |

37. My academic spoken English teacher wholeheartedly using appropriate aids and activities. [Multiple choice] *

| ○1.Never | ○2.Rarely | ○3.Somewhat often | ○4.Often | ○5.Very often |  |  |
| --- | --- | --- | --- | --- | --- | --- |

38. My academic spoken English teacher deals with students’ issues responsibly. [Multiple choice] *

| ○1.Never | ○2.Rarely | ○3.Somewhat often | ○4.Often | ○5.Very often |  |  |
| --- | --- | --- | --- | --- | --- | --- |

39. My academic spoken English teacher gives performance feedback promptly. [Multiple choice] *

| ○1.Never | ○2.Rarely | ○3.Somewhat often | ○4.Often | ○5.Very often |  |  |
| --- | --- | --- | --- | --- | --- | --- |

40.My academic spoken English teacher is not short tempered. [Multiple choice] *

| ○1.Never | ○2.Rarely | ○3.Somewhat often | ○4.Often | ○5.Very often |  |  |
| --- | --- | --- | --- | --- | --- | --- |

41.My academic spoken English teacher likes to joke. [Multiple choice] *

| ○1.Never | ○2.Rarely | ○3.Somewhat often | ○4.Often | ○5.Very often |  |  |
| --- | --- | --- | --- | --- | --- | --- |

42. My academic spoken English teacher is willing to listen to my problems. [Multiple choice] *

| ○1.Never | ○2.Rarely | ○3.Somewhat often | ○4.Often | ○5.Very often |  |  |
| --- | --- | --- | --- | --- | --- | --- |

43. My academic spoken English teacher understands students’ weakness. [Multiple choice] *

| ○1.Never | ○2.Rarely | ○3.Somewhat often | ○4.Often | ○5.Very often |  |  |
| --- | --- | --- | --- | --- | --- | --- |

44. My academic spoken English teacher is approachable even outside classroom. [Multiple choice] *

| ○1.Never | ○2.Rarely | ○3.Somewhat often | ○4.Often | ○5.Very often |  |  |
| --- | --- | --- | --- | --- | --- | --- |

45. My academic spoken English teacher lets me work in my way. [Multiple choice] *

| ○1.Never | ○2.Rarely | ○3.Somewhat often | ○4.Often | ○5.Very often |  |  |
| --- | --- | --- | --- | --- | --- | --- |

46. My academic spoken English teacher speaks gently with students. [Multiple choice] *

| ○1.Never | ○2.Rarely | ○3.Somewhat often | ○4.Often | ○5.Very often |  |  |
| --- | --- | --- | --- | --- | --- | --- |

47. My academic spoken English teacher appreciates my strength and positive results. [Multiple choice] *

| ○1.Never | ○2.Rarely | ○3.Somewhat often | ○4.Often | ○5.Very often |  |  |
| --- | --- | --- | --- | --- | --- | --- |

48. My academic spoken English teacher does not humiliate students. [Multiple choice] *

| ○1.Never | ○2.Rarely | ○3.Somewhat often | ○4.Often | ○5.Very often |  |  |
| --- | --- | --- | --- | --- | --- | --- |

49. My academic spoken English teacher does not show favoritism. [Multiple choice] *

| ○1.Never | ○2.Rarely | ○3.Somewhat often | ○4.Often | ○5.Very often |  |  |
| --- | --- | --- | --- | --- | --- | --- |

50. My academic spoken English teacher assigns responsibility for school related tasks. [Multiple choice] *

| ○1.Never | ○2.Rarely | ○3.Somewhat often | ○4.Often | ○5.Very often |  |  |
| --- | --- | --- | --- | --- | --- | --- |

51. My academic spoken English teacher conducts question and answer session. [Multiple choice] *

| ○1.Never | ○2.Rarely | ○3.Somewhat often | ○4.Often | ○5.Very often |  |  |
| --- | --- | --- | --- | --- | --- | --- |

52. My academic spoken English teacher does not discourage making mistakes. [Multiple choice] *

| ○1.Never | ○2.Rarely | ○3.Somewhat often | ○4.Often | ○5.Very often |  |  |
| --- | --- | --- | --- | --- | --- | --- |

53. My academic spoken English teacher is generous with using encouraging words. [Multiple choice] *

| ○1.Never | ○2.Rarely | ○3.Somewhat often | ○4.Often | ○5.Very often |  |  |
| --- | --- | --- | --- | --- | --- | --- |

54. My academic spoken English teacher encourages students to set high standards for achievement. [Multiple choice] *

| ○1.Never | ○2.Rarely | ○3.Somewhat often | ○4.Often | ○5.Very often |  |  |
| --- | --- | --- | --- | --- | --- | --- |
